# Optimized amino acid ratios in reduced crude protein diets sustain broiler performance, gut health, and resilience to necrotic enteritis

**DOI:** 10.1016/j.aninu.2025.11.011

**Published:** 2026-03-06

**Authors:** Collins A. Asiamah, Sosthene Musigwa, Sarbast K. Kheravii, Alip Kumar, Yadav S. Bajagai, Sara de las Heras-Saldana, Shu-Biao Wu

**Affiliations:** a School of Environmental and Rural Science, University of New England, Armidale 2351, NSW, Australia; b Institute for Future Farming Systems, Central Queensland University, North Rockhampton 4701, Queensland, Australia; c Animal Genetics and Breeding Unit, A Joint Venture of NSW Department of Primary Industries and Regional Development and University of New England, Armidale 2351, NSW, Australia

**Keywords:** Necrotic enteritis, Essential-to-total amino acid ratio, Reduced crude protein, Broiler

## Abstract

This study investigated the effects of essential-to-total amino acid (E:T) ratios in reduced crude protein (CP) diets on broiler performance, gene expression, and cecal microbiota under necrotic enteritis (NE) challenge. A total of 756 mixed-sex 0-d-old Cobb 500 broilers were assigned to six treatments in a 2 × 3 factorial arrangement, with two challenge conditions (yes or no) and three dietary treatments: 17% reduced CP (RCP) with E:T ratio of 0.64 (RCP0.64), 17% RCP with E:T ratio of 0.68 (RCP0.68), and 19% normal CP as control (NCP) from d 9 to 18. Performance was measured from d 8 to 18 post–hatch, while jejunal, spleen, and cecal samples were collected on d 16 for gene expression and microbiota analysis. Results showed a significant interaction between diet and challenge for weight gain (WG; *P* = 0.004) and feed intake (FI; *P* = 0.007). Among unchallenged birds, those fed the RCP0.64 diet achieved WG and FI comparable to control-fed birds and were significantly higher than those fed the RCP0.68 diet (*P* < 0.001). Under the NE challenge, FI was significantly higher in the RCP0.64 group than in the control group (*P* < 0.001). Both RCP diets and NE challenge increased the feed conversion ratio (*P* < 0.001). Gene expression analysis showed a diet × challenge interaction for *IgG* (jejunum, *P* = 0.045) and *IgM* (spleen, *P* = 0.042). The NE challenge increased *IgM* expression only in RCP0.64-fed birds (*P* = 0.012). The main effects showed that the NE challenge upregulated nutrient transporter, immune, and apoptotic genes and downregulated *TJP1* and *GLUT2* (*P* < 0.001) in the jejunum. In the spleen, *IL2* and *IL6* expressions were significantly lower (*P* < 0.05) in RCP0.64-fed birds than in those fed a control diet. In RCP0.64-fed birds, microbiota differential analysis revealed higher Firmicutes in unchallenged birds but increased pathogenic *Clostridium sensu stricto 1* in challenged birds. These findings demonstrate that the RCP0.64 diet can maintain WG and FI in healthy birds and partially preserve gut health during NE challenge by modulating immune responses (*IgG* and *IgM*) and microbial communities. This dietary strategy could be effective for reducing protein in sustainable poultry production.

## 1 Introduction

Optimizing the nutritional efficiency of broiler diets is crucial for achieving sustainable poultry production while addressing challenges such as feed cost, environmental impact, and disease management. Protein is a major component of broiler diets, directly influencing growth, immunity, and gut health. However, conventional dietary crude protein (CP) levels can increase feed costs and contribute to excess nitrogen excretion, which is a global environmental concern (Belloir et al., 2017; Castanheira and Freire, 2013; da Silva et al., 2010). This has led poultry researchers to focus on reduced crude protein (RCP) diets supplemented with essential amino acids (EAA), depending on the degree of CP reduction, to maintain performance and decrease nitrogen excretion.

Formulating RCP diets requires a balanced supply of EAA and non-essential amino acids (NEAA) to obtain the total amount of nitrogen for optimal performance (Camiré et al., 2023; Musigwa et al., 2024). However, most research has focused on meeting EAA requirements, overlooking the fact that NEAA contribute nearly half of the total dietary nitrogen (Heger et al., 1998). Although poultry can endogenously synthesize NEAA from excess EAA, an imbalance between these two groups may limit protein utilization efficiency and overall performance (Asiamah et al., 2025; Musigwa et al., 2024). The essential-to-total amino acid (E:T) ratio is a promising strategy to ensure AA balance and an adequate supply of EAA and NEAA (Alhotan and Pesti, 2016; Heger, 2003), which may optimize EAA utilization and thus nitrogen retention (Camiré et al., 2023). Previous studies suggested E:T ratios ranging from 0.55 to 0.60 as ideal for optimizing growth in poultry, pigs, and rats (Heger, 2003). Recently, Musigwa et al. (2024) and Asiamah et al. (2025), respectively, reported that the E:T ratio of 0.60 (excluding Tyr as EAA) or 0.64 (including Tyr as EAA) in RCP diets enhanced nutrient utilization and maintained performance comparable to that of normal protein-fed broiler chickens.

Beyond nutritional factors, necrotic enteritis (NE), caused by *Clostridium perfringens*, remains a significant health challenge, which impairs performance, increases mortality, and results in substantial financial losses in poultry production (Wade and Keyburn, 2015). The NE disrupts gut integrity, alters microbiota composition, triggers inflammatory responses, and impairs nutrient absorption and feed efficiency (Gharib-Naseri et al., 2020; Goo et al., 2024; Keerqin et al., 2021). While many dietary additives, such as probiotics, have emerged as potential alternatives to antibiotics for NE mitigation (Gharib-Naseri et al., 2020; Keerqin et al., 2021), dietary modifications, such as optimizing the E:T ratio in RCP diets, could offer additional benefits. An optimized E:T ratio RCP diet could maximize nutrient utilization, reduce nitrogen excretion (Camiré et al., 2023; Musigwa et al., 2024), and potentially limit nitrogen availability and excessive undigested protein substrates in the hindgut, a substrate for *C*. *perfringens* proliferation, thus NE development (Jianfei et al., 2019; Paiva and McElroy, 2014). However, the potential effect of an optimized E:T ratio in RCP diets on mitigating the impact of NE remains largely unexplored.

The jejunum serves as the primary site for nutrient absorption, immune defense, and intestinal barrier maintenance (Sinpru et al., 2021; Zhu et al., 2025), whereas the spleen is a major organ for systemic immune responses (Zhang et al., 2019). Changes in the expression of nutrient transporters, tight junction proteins, mucin, and inflammatory cytokines in these tissues provide insights into the impact of dietary and disease stressors on broiler physiology (Gharib-Naseri et al., 2020; Jia et al., 2024). Additionally, the gut microbiota plays a vital role in nutrient utilization, immune function, and overall performance in broilers, with microbial imbalances often linked to disease and poor productivity (Aruwa and Sabiu, 2024; Barathan et al., 2024). Several studies have shown that NE significantly downregulates nutrient transporters and tight junction proteins (Gharib-Naseri et al., 2021; Goo et al., 2024, 2023), alters immune response, and increases cell apoptosis in the jejunum and spleen (Gharib-Naseri et al., 2020; Kulkarni et al., 2023), while causing dysbiosis in the cecal gut microbiota (Moore, 2024). However, there is a lack of studies evaluating the impact of varied E:T ratios in RCP diets on the jejunal and splenic gene expression and cecal microbiota under NE challenge.

This study aimed to evaluate the effects of RCP diet with varying E:T ratios on performance, gene expression patterns in the jejunum and spleen, and cecal microbiota composition in broilers under NE challenge. This study hypothesized that an optimal E:T ratio in RCP diets mitigates the adverse effects of NE on performance, enhances expression of nutrient transporters, supports gut barrier integrity and immunity, and modulates gut microbiota. It was also hypothesized that an optimized E:T ratio in the RCP diet positively affects birds at a level similar to that of normal CP-fed birds. The findings provide valuable insights into dietary strategies that support sustainable poultry production under health-challenging conditions.

## 2 Materials and methods

### 2.1 Animal ethics statement

The Animal Ethics Committee of the University of New England approved the animal experimental protocols described in this study (approval number ARA22–073).

### 2.2 Experimental design

The experiment followed a 2 × 3 factorial arrangement of treatments, incorporating two factors: NE challenge (yes [Y] or no [N]) and diet type at three levels. The dietary factor included: RCP diet with an E:T ratio of 0.64 (RCP0.64), RCP diet with an E:T ratio of 0.68 (RCP0.68), and normal CP control diet (NCP; 19% CP). As NCP differed in CP content, it was included as a control rather than as part of a fully orthogonal factorial structure. Both RCP diets (RCP0.64 and RCP0.68) contained 17% CP for the grower stage. This yielded six treatment combinations (Table 1), with each treatment replicated seven times, and 18 birds were assigned to each replicate.

**Table 1:** Treatment design.

Groups	NE challenge	Diets^1^	CP, %	E:T ratio
1	No	RCP0.64	17	0.64
2	No	RCP0.68	17	0.68
3	No	NCP	19	0.53
4	Yes	RCP0.64	17	0.64
5	Yes	RCP0.68	17	0.68
6	Yes	NCP	19	0.53

CP = crude protein; E:T ratio = essential-to-total amino acids ratio; NE = necrotic enteritis; RCP = reduced crude protein; NCP = normal crude protein.

1 RCP0.64, 17% CP with an E:T ratio of 0.64; RCP0.68, 17% CP with an E:T ratio of 0.68; NCP, 19% CP.

### 2.3 Diet preparation

The composition and calculated nutrient content of the experimental diets are provided in Table 2, Table 3, respectively. All diets were based on wheat, barley, soybean meal, and corn and were formulated to be isoenergetic on a net energy basis across treatments. The main dietary nutrients were calculated using near-infrared spectroscopy (NIRS). The diets were designed to meet the nutrient specifications for Cobb 500 (2022a), except for the RCP grower diets (RCP0.64 and RCP0.68), which contained 17% CP. Phytase and multi-carbohydrase enzymes were supplemented in diets according to their respective manufacturer-recommended nutrient matrix values. The E:T ratio was used as a tool to balance NEAA. The RCP and NCP diets were isonitrogenous, containing 17% and 19% CP, respectively (total nitrogen × 6.25). Thus, any changes in the E:T value occurred while maintaining a constant CP level of 17% and EAA levels across the two RCP diets. The optimal E:T ratios were achieved following previous studies by adjusting NEAA as mentioned above (Musigwa et al., 2024). The feed formulation process considered both EAA and NEAA to achieve a balanced ratio E:T. Total AA (TAA; including EAA and NEAA) content was calculated using the method described by Alhotan and Pesti (2016):

Feedproteincontent(%)=Ingredientprotein(%)×Ingredientamount(%);

Ingredienttotalnitrogen(%)=CPcontent/6.25;

$$\text{Ingredient}\;\text{TAA}\;\text{contribution}\;\left(\%\right)\;\text{to}\;\text{feed}=\text{Ingredient}\;\text{total}\;\text{nitrogen}\times K_{A}\text{;}$$

where K_A_ is the specific nitrogen-to-protein conversion factor for each ingredient (Krul, 2019; Mosse, 1990). In addition, Gly equivalent (equiv; %) was calculated as:

Glyequiv(%)=Gly(%)+[0.7143×Ser(%)],

where 0.7143 is the ratio of the molar weight between Gly and Ser (Chrystal et al., 2018). Dietary electrolyte balance (DEB; mEq/kg) was calculated as (Chrystal et al., 2020):

DEB(mEq/kg)=(Na÷0.0023)+(K÷0.00391)–(Cl÷0.00355).

**Table 2:** Ingredient composition of diets used (%, as is basis).

Ingredients	Starter (d 0–8)	Grower (d 9–18)^4^		
		RCP0.64	RCP0.68	NCP
Wheat	30.000	10.000	13.000	20.500
Barley	10.900	28.600	25.700	30.000
Soybean meal	29.800	10.400	7.900	22.200
Wheat pollard	1.000	8.200	15.200	1.000
Corn	10.000	15.000	13.000	10.000
Sorghum	3.900	1.000	1.000	9.200
Canola meal solvent	4.000	7.200	7.180	0.250
Canola oil	4.170	6.150	6.000	2.440
Sawdust	0.940	2.500	1.190	0.000
Diatomaceous earth	0.000	2.500	1.190	0.000
Na bentonite	2.000	2.000	2.000	0.000
Carbohydrases^1^	0.005	0.005	0.005	0.005
Phytases^2^	0.010	0.010	0.010	0.010
K carbonate	0.000	0.562	0.553	0.255
Limestone	1.329	1.208	1.229	1.290
Monocalcium P	0.715	0.666	0.617	0.583
Salt	0.299	0.127	0.102	0.220
Na bicarbonate	0.000	0.186	0.219	0.054
TiO2	0.000	0.500	0.500	0.500
Vitamins^3^	0.070	0.070	0.070	0.070
Trace minerals^3^	0.100	0.100	0.100	0.100
Choline chloride (70%)	0.086	0.134	0.146	0.065
L-Lys HCl (78.4%)	0.254	0.669	0.722	0.401
DL-Met	0.251	0.242	0.249	0.206
L-Thr	0.066	0.278	0.303	0.140
L-Arg free base	0.000	0.424	0.459	0.161
L-Val	0.064	0.296	0.318	0.136
L-Ile	0.000	0.267	0.298	0.087
L-Leu	0.000	0.326	0.381	0.000
L-Phe	0.000	0.098	0.127	0.000
L-Cys	0.000	0.132	0.136	0.089
L-Gly	0.000	0.135	0.161	0.000
Total	100.000	100.000	100.000	100.000

CP = crude protein; RCP = reduced crude protein; NCP = normal crude protein.

1 Rovabio Advance T-Flex (xylanase, β-glucanase, and arabinofuranosidase).

2 AXTRA PHY Gold 10T (Dupont Animal Nutrition) provided 500 phytase units (FTU)/kg.

3 Vitamin and mineral concentrate supplied per kg diet: 5040 mg retinol, 17.5 mg cholecalciferol, 105 mg tocopheryl acetate, 4 mg menadione, 4 mg thiamine, 11 mg riboflavin, 77 mg niacin, 18 mg pantothenate, 7 mg pyridoxine, 0.35 mg biotin, 3.0 mg folate, 0.02 mg cyanocobalamin, 23 mg copper, 1.79 mg iodine, 57 mg iron, 171 mg manganese, 0.43 mg selenium and 143 mg zinc.

4 RCP0.64, 17% CP with an E:T ratio of 0.64; RCP0.68, 17% CP with an E:T ratio of 0.68; NCP, 19% CP.

**Table 3:** Calculated dietary nutrient composition (%, as is basis).

Nutrients	Starter (d 0–8)	Grower (d 9–18)^1^		
		RCP0.64	RCP0.68	NCP
ME, MJ/kg	12.69	12.66	12.66	12.86
Net energy, MJ/kg	9.86	10.03	10.03	10.03
CP	22.00	17.00	17.00	19.00
Crude fat	5.72	7.70	7.59	4.07
Crude fiber	3.98	5.95	5.68	3.82
Total EAA	10.14	8.65	8.65	8.95
TP	19.73	14.42	13.52	17.05
Gly equiv	1.391	1.000	1.000	1.120
Digestible Arg	1.280	1.176	1.176	1.176
Digestible Lys	1.220	1.120	1.120	1.120
Digestible Met	0.544	0.448	0.448	0.448
Digestible Cys	0.336	0.358	0.358	0.358
Digestible Met + Cys	0.880	0.806	0.806	0.806
Digestible Trp	0.286	0.182	0.179	0.237
Digestible His	0.502	0.326	0.314	0.411
Digestible Phe	0.933	0.672	0.672	0.777
Digestible Leu	1.457	1.221	1.221	1.221
Digestible Ile	0.817	0.750	0.750	0.750
Digestible Tyr	0.648	0.396	0.382	0.535
Digestible Asn	0.765	0.450	0.425	0.606
Digestible Thr	0.817	0.750	0.750	0.750
Digestible Val	0.939	0.862	0.862	0.862
Digestible Gly	0.753	0.625	0.643	0.600
Digestible Ser	0.892	0.525	0.500	0.728
Digestible Pro	1.180	0.872	0.868	1.081
Digestible Ala	0.811	0.535	0.520	0.688
Digestible Asp	1.147	0.614	0.575	0.907
Digestible Glu	2.543	1.341	1.337	2.044
Digestible Gln	2.212	1.474	1.481	1.869
Starch	33.01	34.37	35.84	40.83
Ca	0.880	0.800	0.800	0.800
Available P	0.440	0.400	0.400	0.400
Sodium	0.177	0.160	0.160	0.160
Potassium	0.950	0.950	0.950	0.950
Chloride	0.300	0.300	0.300	0.300
Choline, mg/kg	1700	1500	1500	1500
Linoleic 18:2	1.966	2.378	2.325	1.575
DEB (Na + K–Cl)	235.000	228.000	228.000	228.000
E:T ratio	0.510	0.640	0.680	0.530

ME = metabolizable energy; CP = crude protein; TP = true protein; EAA = essential amino acids; equiv = equivalent; DEB = dietary electrolyte balance; E:T ratio = essential-to-total amino acids ratio; RCP = reduced crude protein; NCP = normal crude protein.

1 RCP0.64, 17% CP with an essential-to-total amino acid (E:T) ratio of 0.64; RCP0.68, 17% CP with an E:T ratio of 0.68; NCP, 19% CP.

The analyzed concentrations of CP and AA in the experimental diets are shown in Table 4.

**Table 4:** Analyzed nutrient contents in experimental diets (%, as is basis).

Nutrients	Grower (d 9–18)^1^		
	RCP0.64	RCP0.68	NCP
His	0.366	0.334	0.479
Ser	0.621	0.568	0.874
Arg	1.123	1.092	1.222
Gly	0.737	0.722	0.767
Asp	1.111	0.957	1.666
Glu	2.695	2.573	3.563
Thr	0.740	0.746	0.787
Ala	0.588	0.538	0.810
Pro	0.952	0.919	1.178
Lys	1.187	1.136	1.204
Tyr	0.294	0.266	0.463
Met	0.337	0.359	0.397
Val	0.889	0.853	0.972
Ile	0.809	0.799	0.843
Leu	1.336	1.310	1.429
Phe	0.726	0.700	0.894
CP	16.37	15.97	18.50

CP = crude protein; RCP = reduced crude protein; NCP = normal crude protein.

1 RCP0.64, 17% CP with an essential-to-total amino acid (E:T) ratio of 0.64; RCP0.68, 17% CP with an E:T ratio of 0.68; NCP, 19% CP.

### 2.4 Animal management and NE challenge

A total of 756 mixed-sex 0-d-old Cobb 500 broilers were obtained from Baiada Hatchery (Tamworth, NSW, Australia). Upon arrival, the chicks were weighed and randomly assigned to 42-floor pens with 18 birds per pen. They were fed in two phases ad libitum: a common crumbled starter diet (d 0–8) followed by pelleted treatment grower diets from d 9 to 18. In an environmentally controlled facility, birds were managed following Cobb 500 (2022b) management guidelines using softwood shavings as litter material.

The NE challenge followed a modified protocol based on Rodgers et al. (2015). Briefly, on d 9, all 378 challenged birds received 1 mL oral inoculation of live vaccine of *Eimeria* spp., consisting of 5000 oocysts of each *E. acervulina* and *E*. *maxima*, along with 2500 oocysts of *E. brunetti* (Eimeria Pty Ltd., Werribee, VIC, Australia). The 378 unchallenged birds received a sham treatment of 1 mL sterile phosphate-buffered saline to mimic inoculation stress. On d 14 and 15, the challenged birds were orally administered a dose of 1 mL solution containing 1 × 10^8^ colony forming unit (CFU)/mL of the *C. perfringens* EHE-NE18 strain (CSIRO Livestock, Geelong, VIC, Australia). Birds in the non-challenge groups received 1 mL sterile thioglycollate broth media as a sham treatment.

### 2.5 Performance measurement and sample collection

Birds and feed were weighed on d 8 and 19 to calculate feed intake (FI) and weight gain (WG). The weight of dead and sampled birds was recorded to correct the feed conversion ratio (FCR) for mortality. On d 16, four birds per pen (2 males and 2 females) were randomly selected and euthanized by electrical stunning followed by cervical dislocation. Jejunum and spleen tissues were collected immediately from one male bird per pen (7 birds per treatment). The tissues were quickly rinsed with sterile phosphate-buffered saline (PBS), placed in RNA later, and kept at 4 °C for 4 h before being stored at −20 °C for RNA extraction. The entire gastrointestinal tract of the sampled birds was dissected, and the cecum was isolated. Subsequently, cecal digesta was gently collected from all four birds and pooled into a sterile 60-mL container. After thorough mixing, aliquots were transferred into 2-mL Eppendorf tubes (filled to two-thirds capacity) to prevent leakage during freezing. The tubes were immediately snap-frozen in liquid nitrogen to preserve sample integrity and were later stored at −20 °C until further processing.

### 2.6 Chemical analysis and calculations

Diets and digesta samples were dried at 105 °C in a Westlab Incu-Oven, 56 L (code 663–913, Westlab, Mitchell Park, VIC, Australia) until a consistent weight was reached for dry matter (DM) analysis. The dietary AA contents were analyzed using the Waters AccQTag AA method adapted for ultra performance liquid chromatography (UPLC), with an ACQUITY UPLC system and UV detector (Waters Corp., Milford, MA, USA) (Bosch et al., 2006).

The freeze-dried and ground excreta and feed samples were analyzed for their gross energy (GE) using an adiabatic bomb calorimeter (Parr 6400 automatic isoperibol calorimeter, Parr Instrument Co., Moline, IL, USA). Additionally, nitrogen content of each diet was determined by the Dumas combustion method (Sweeney, 1989) using a Leco TruMac CNS analyser (Leco Corp., St. Joseph, MI, USA). Then CP was calculated by multiplying the total nitrogen obtained by the standard nitrogen conversion factor of 6.25.

The concentration of TiO_2_ in diets and digesta samples was evaluated according to the procedure outlined by Short et al. (1996). Feed net energy was calculated according to Noblet et al. (1994). Feed metabolizable energy (ME; MJ/kg DM) was calculated using TiO_2_ as the indigestible marker, based on Scott and Hall (1998) methodology as follows:

$$\text{ME}\;\left(\text{MJ}/\text{kg}\;\text{DM}\;\text{of}\;\text{diet}\right)=\text{Diet}\;\text{GE}\;–\;\left[\text{Excreta}\;\text{GE}\;\left(\text{Diet}\;{\text{TiO}}_{2}/\text{Excreta}\;{\text{TiO}}_{2}\right)\right]\text{.}$$

### 2.7 RNA extraction and complementary DNA (cDNA) synthesis

Following the manufacturer’s instructions, RNA was extracted from forty-two of each of the jejunum and spleen tissues (7 birds/treatment) using Isolate II RNA Mini Kit (Bioline, Meridian Bioscience, Sydney, NSW, Australia). RNA concentration and purity were measured with a NanoDrop ND-8000 spectrophotometer (Thermo Fisher Scientific Inc., Waltham, MA, USA), and RNA integrity was assessed with an Agilent 2100 Bioanalyzer using RNA 6000 Nano Kit (Agilent Technologies, Inc., Waldron, Germany). RNA samples with A_260/280_ ratios ranging from 1.8 to 2.2, and an RNA integrity number (RIN) > 6.0 were considered of high quality. The concentrations of the high-quality RNA samples were diluted using RNAse-free water to 100 ng/μL before they were reverse transcribed. cDNA was synthesized using a SensiFast cDNA synthesis kit (Bioline, Meridian Bioscience, Sydney, NSW, Australia) according to the manufacturer’s protocol on a Rotor-Gene Q real-time PCR machine (Qiagen GmbH, Hilden, Germany). cDNA solution was diluted 10 times with nuclease-free water and stored at −20 °C until required for real-time quantitative PCR (RT-qPCR).

### 2.8 Real-time quantitative PCR

Primers targeting specific genes of interest related to nutrient transporters, immunity, cell apoptosis, mucin production, and tight junctions were previously designed within the laboratory or sourced from literature, as shown in Table 5. Notably, a typographical error in the reverse primer sequence for *CASP8* reported in previous study (Gharib-Naseri et al., 2021) has been corrected in this work. The SensiFAST SYBR No-ROX kit (Meridian Bioscience, Sydney, NSW, Australia) and a Rotor-Gene Q real-time PCR machine (Qiagen GmbH, Hilden, Germany) were used to quantify gene expression using two replicates per sample. The PCR amplification was performed in a 10 μL reaction volume containing 5 μL 2 × SensiFAST, 0.4 μL of each primer, 2.2 μL nuclease-free water, and 2 μL 10 × diluted cDNA template. The reaction conditions involve an initial denaturation at 95 °C for 2 min, followed by 40 cycles of denaturation at 95 °C for 5 s and annealing at the respective temperatures (Table 5) for 20 s. The fluorescent data were acquired at the end of each PCR cycle and a melting step (55–99 °C, increasing by 0.5 °C per step, with a pre-melt conditioning hold of 90 s on the first step and a 5 s hold at each step afterwards) was conducted to assess the specificity of PCR amplification. The gene expression stability measurement (geNorm M) of eight widely used housekeeping genes was calculated to select the two most stable genes using qBase + version 3.0 (Biogazelle, Zwijnbeke, Belgium). The assessed housekeeping genes were *β-ACT* (the full names of the abbreviations of all genes can be found in Table S2), *GAPDH*, *RPL4*, *TBP*, *HPRT1*, *HMBS*, *YWHAZ*, and *SDHA*.

**Table 5:** Sequences of primers used for quantitative real-time PCR.

Genes	Accession No.	Sequences (5'-3')	Size, bp	Ta, ^o^C	Reference
**Reference genes**					
*RPL4*	NM_001007967	F: TTATGCCATCTGTTCTGCC	235	60	Yang et al. (2013)
		R: GCGATTCCTCATCTTACCCT			
*β-ACT*	AB495656.1	F: CTCTGACTGACCGCGTTACTCC	175	60	Zanu et al. (2020b)
		R: CCATACCAACCATCACACCCTG			
*SDHA*	NM_001277398.1	F: ATACGGGAAGGAAGGGGTTG	74	60	Barzegar et al. (2021)
		R: TGCTGGGGTGGTAAATGGTG			
**Nutrient transporter genes**					
*ASCT*1	XM 001232899	F: TTGGCCGGGAAGGAGAAG	63	60	Paris and Wong (2013)
		R: AGACCATAGTTGCCTCATTGAATG			
*B*^*0*^*AT*	XM_419056.5	F: GTGTTTGGAACCCTAAATACGAGG	72	60	Kheravii et al. (2018)
		R: TAGCATAGACCCAGCCAGGA			
*b*^*0,+*^*AT*	NM_001199133.1	F: CAGTAGTGAATTCTCTGAGTGTGAAGCT	88	60	Gilbert et al. (2007)
		R: GCAATGATTGCCACAACTACCA			
*GLUT2*	NM_207178.1	F: TGATCGTGGCACTGATGGTT	171	60	Kheravii et al. (2018)
		R: CCACCAGGAAGACGGAGATA			
*LAT1*	KT876067.1	F: GATTGCAACGGGTGATGTGA	70	60	Gilbert et al. (2007)
		R: CCCCACACCCACTTTTGTTT			
*PepT1*	AY029615.1	F: TACGCATACTGTCACCATCA	205	60	Guo et al. (2014)
		R: TCCTGAGAACGGACTGTAAT			
**Tight junction genes**					
*TJP1*	XM_413773.4	F: GGATGTTTATTTGGGCGGC	187	60	Zanu et al. (2020a)
		R: GTCACCGTGTGTTGTTCCCAT			
*JAM2*	XM_046907882.1	F: AGACAGGAACAGGCAGTGCTAG	135	60	Zanu et al. (2020b)
		R: ATCCAATCCCATTTGAGGCTAC			
*OCLD*	NM_205128.1	F: ACGGCAGCACCTACCTCAA	123	60	Du et al. (2016)
		R: GGGCGAAGAAGCAGATGAG			
**Mucin production gene**					
*MUC2*	XM_001234581.3	F: CCCTGGAAGTAGAGGTGACTG	143	60	Fan et al. (2015)
		R: TGACAAGCCATTGAAGGACA			
**Immunity genes**					
*IFNY*	NM_205149.1	F: AGCTGACGGTGGACCTATTATT	259	60	Li et al. (2018)
		R: GGCTTTGCGCTGGATTC			
*IL2*	NM_204153.1	F: TCTGGGACCACTGTATGCTCT	256	60	Liu et al. (2018)
		R: ACACCAGTGGGAAACAGTATCA			
*IL6*	AJ250838	F: GCTCGCCGGCTTCGA	188	60	Smith et al. (2005)
		R: GGTAGGTCTGAAAGGCGAACAG			
*IgA*	S40610.1	F: GTCACCGTCACCTGGACTACA	192	64	Lammers et al. (2010)
		R: ACCGATGGTCTCCTTCACATC			
*IgG*	X07174.1	F: ATCACGTCAAGGGATGCCCG	118	60	Lammers et al. (2010)
		R: ACCAGGCACCTCAGTTTGG			
*IgM*	X01613.1	F: GCATCAGCGTCACCGAAAGC	98	60	Lammers et al. (2010)
		R: TCCGCACTCCATCCTCTTGC			
Cell apoptosis genes					
*CASP3*	NM_204725.1	F: TGGTGGAGGTGGAGGAGC	110	62	Gharib-Naseri et al. (2021)
		R: GTTTCTCTGTATCTTGAAGCACCA			
*CASP8*	NM_204592.4	F: GGAGCTGCTCTATCGGATCAAT	126	60	Gharib-Naseri et al. (2021)
		R: AGCAGATACCTGAACGGAGACAC			

F = forward; R = reverse; bp = base pairs; Ta = annealing temperature.

The cycle threshold values of the target genes were analyzed against two optimized reference genes selected for each tissue (*ACTB* and *RPL4* for jejunum samples and *ACTB* and *SDHA* for spleen samples) using qBase + version 3.0 (Biogazelle, Wijnbeke, Belgium). The qBase + software applied an arithmetic mean method to transform the logarithmic value of the amplification cycle to a linear relative quantity using the exponential function for the relative quantification of target genes. Expression levels of the genes were expressed as the means of relative normalized amounts of genes (Hellemans et al., 2007; Vandesompele et al., 2002) in respective treatments.

### 2.9 DNA extraction and 16S rRNA sequencing

The DNA was extracted from 42 cecal excreta samples using the DNeasy 96 PowerSoil Pro QIAcube HT Kit (Qiagen, Inc., Doncaster, VIC, Australia) according to the manufacturer’s instructions. Approximately 60 mg frozen samples were weighed in a 2-mL Eppendorf tube with 300 mg glass beads (0.1 mm diameter, Daintree Scientific, St Helens, Tasmania, Australia). Then, 700 μL CD1 solution was added, vortexed for 5 s, and placed into a Tissuelyser II (Qiagen GmbH, Hilden, Germany) for 5 min at a frequency of 30 Hz to disrupt the bacterial cells. The samples were briefly spun and incubated on a heat block at 90 °C for 10 min, followed by vortexing for 5 s and centrifuging for 1 min at 15,000 × *g*. Then, approximately 600 μL supernatant was transferred to a new 2-mL Eppendorf tube. Then, 250 μL CD2 was added to the samples, vortexed briefly, and centrifuged at 4500 × *g* for 5 min. The supernatant was transferred to a new S-Block and placed in the correct position in QIAcube HT (Qiagen GmbH, Hilden, Germany) to complete the extraction process according to the manufacturer’s specifications. The extracted DNA concentration and quality were measured using a NanoDrop ND-8000 spectrophotometer. DNA samples with A_260/A280_ ratios greater than 1.8 were considered of high quality and stored at −20 °C until required.

DNA samples were diluted in nuclease-free water to a target final concentration of 20 ng/μL. The 16S rRNA gene specific to the 16S V3/V4 regions was amplified using the Pro341F-Pro805R primer set, which included barcodes, heterogeneity spacer sequences, Illumina sequencing linkers, and Q5 high-fidelity 2 × master mix (New England Biolab, Notting Hill, VIC, Australia). Nuclease-free water was used as a negative control for each PCR amplification. The amplified PCR product was purified using Invitrogen E-Gel SizeSelect II Agarose Gels (Thermo Fisher Scientific Inc., Scoresby, VIC, Australia) and AMPure XP bead-based reagent (Beckman Coulter, Lane Cove, NSW, Australia), following the manufacturer’s protocols. The quality of the purified library was analyzed with 2100 Bioanalyzer (Agilent Technologies Inc., Santa Clara, CA, USA) and quantified using Qbit and RT-qPCR. DNA sequencing was performed with 30% PhiX spike-in using the Illumina MiSeq platform (Illumina Inc., San Diego, CA, USA), resulting in 300 bp paired-end reads.

### 2.10 Bioinformatics in microbiota analysis

The quality of the raw sequencing data was assessed using Fast QC v0.11.2 (Andrews, 2010) and MultiQC v1.11 (Ewels et al., 2016) to evaluate the read quality and identify potential issues such as adapter contamination, sequence duplication, and base-call accuracy. The sequencing primers were removed with Cutadapt (Martin, 2011). The filtering, denoising, and chimera removal of the sequencing data were performed using Quantitative Insights into Microbial Ecology 2 (QIIME2 v2023.7) (Bolyen et al., 2019). Specifically, the DADA2 plugin implemented in QIIME2 was utilized for quality filtering, denoising, and chimeric sequence removal (Callahan et al., 2016). The denoised amplicon sequence variants (ASVs) were assigned a taxonomic classification based on the reference database SILVA v138 (Quast et al., 2013).

Downstream analysis and visualization of the microbiota composition and diversity were conducted with R packages, including Phyloseq v1.50.0 (McMurdie and Holmes, 2013) and microeco v1.14.0 (Liu et al., 2021). Alpha diversity indices (Shannon, abundance-based coverage estimator [ACE], Chao1, and Simpson) were used to measure species richness, evenness, and diversity within individual samples. Differences in alpha diversity among groups were analyzed with the non-parametric Kruskal–Wallis tests. Beta diversity metrics, specifically unweighted and weighted UniFrac distances, were used to evaluate differences in microbial community between groups based on phylogenetic information (Lozupone and Knight, 2005; Lozupone et al., 2007). For statistical analysis of UniFrac distances, the non-parametric permutational analysis of variance (PERMANOVA) test was employed. Principal coordinates analysis (PCoA) was used to visualize distances between samples, where clustering of points indicated similarities between microbial communities within treatment groups. The ggplot2 v.3.5.1 (Wickham, 2016) package in R was used to visualize box plots and a PCoA scatter plot. Linear discriminant analysis effect size (LEfSe) was performed to identify differentially abundant phyla and genera across groups (Segata et al., 2011). Taxa with a linear discriminant analysis (LDA) score > 2.0 were considered potential biomarkers.

### 2.11 Statistical analysis

The analyses were done with R software v.4.1.2 (R Core Team, 2021). Initially, the normality and homogeneity of variance were assessed for the performance and gene expression (means of relative normalized count of genes) data using the Shapiro–Wilk normality test and Levene’s homogeneity test, respectively. Outliers were identified and removed using the interquartile range (IQR) method:

$$\text{IQR}\;\left(x\right)=\text{Quantile}\;\left(x,3/4\right)\;–\;\text{Quantile}\;\left(x,1/4\right)\text{,}$$

where based on the quantile function in R. Data points below Q1 – 1.5 × IQR or above Q3 + 1.5 × IQR were considered outliers and excluded from further analysis (Tukey, 1977).

Performance and gene expression data were analyzed using a completely randomized design in a 2 × 3 factorial arrangement, with the percentage of male birds serving as a covariate to evaluate the main effects and interactions of diet and challenge. Analyses were conducted using R software v.4.1.2 (R Core Team, 2021). Pen was the experimental unit, and the linear mixed model (LMM) for the analysis was:

$$Y_{ijk}=\mu +\alpha_{i}+\beta_{j}+{\left(\alpha \beta \right)}_{ij}+u_{k}+\varepsilon_{ijk}\text{,}$$

where *Y*_*ijk*_ is the response variable (performance parameters and gene expression); *μ* is the overall mean; *α*_*i*_ is the fixed effect of *i-*th diet (RCP0.64, RCP0.68, and NCP); *β*_*j*_ is the fixed effect of challenge condition (challenged, unchallenged); (*αβ*)_*ij*_ is the fixed effect of interaction between diet and challenge; *u*_*k*_ is the random effect of the *k-*th experimental unit; *ε*_*ijk*_ is the residual error term, assumed *ε*_*ijk*_ ∼ N (0,σ^2^).

The analysis was performed using the estimated marginal means obtained from the emmeans function in the emmeans R package v.1.10.4 to assess the associations between the independent variables (diet and challenge) and the dependent variables (performance and gene expression). Post-hoc multiple comparisons among treatment means were conducted using Tukey’s Honest Significant Difference (HSD) test in the estimated marginal means (emmeans) package (v.1.10.4), with the compact letter display (cld) function used to separate significant differences. Statistical significance was set at a threshold of *P* < 0.05.

## 3 Results

### 3.1 Effects of diet and NE challenge on broiler performance at the grower stage

Table 6 presents the effects of diet and challenge on broiler performance during d 8 and 19. A significant interaction between diet and NE challenge was observed for WG (*P* = 0.004) and FI (*P* = 0.007). Among unchallenged birds, those fed RCP0.64 and NCP had significantly higher WG (*P* < 0.001) compared to birds fed RCP0.68, with no significant difference (*P* = 1.000) observed between RCP0.64 and NCP-fed birds. However, within the challenged group, no significant differences in WG were observed among the three dietary treatments (*P* > 0.05). Regarding FI, in unchallenged birds, RCP0.64-fed birds consumed significantly more feed (*P* < 0.001) than those on RCP0.68, while in challenged birds, RCP0.64-fed birds had significantly higher FI (*P* < 0.001) than those fed NCP.

**Table 6:** Impact of diet and NE challenge on performance at the grower stage (d 8–19).

Items	Factors		WG, g/bird per d	FI, g/bird per d	FCR
Groups	Challenge	Diet^1^			
1	N	RCP0.64	63.6^a^	82.6^a^	1.298
2	N	RCP0.68	57.2^b^	75.5^bc^	1.322
3	N	NCP	63.6^a^	78.6^ab^	1.235
4	Y	RCP0.64	55.1^bc^	78.5^ab^	1.424
5	Y	RCP0.68	52.8^c^	75.4^bc^	1.428
6	Y	NCP	53.2^c^	71.3^c^	1.340
Main effects	Diet	RCP0.64	59.4	80.5	1.361^a^
		RCP0.68	55.0	75.5	1.375^a^
		NCP	58.4	74.9	1.288^b^
	Challenge	N	61.5	78.9	1.285
		Y	53.7	75.1	1.397
SEM			0.77	0.68	0.0110
*P-*value	Diet		<0.001	<0.001	<0.001
	Challenge		<0.001	<0.001	<0.001
	Diet × challenge		0.004	0.007	0.231
	Sex covariate		ns	ns	ns

CP = crude protein; NE = necrotic enteritis; N = no NE challenge; Y = NE challenge; SEM = standard error of mean; WG = weight gain; FI = feed intake; FCR = feed conversion ratio; ns = non-significant; RCP = reduced crude protein; NCP = normal crude protein.

Within a column, diet × challenge means without a common superscript letter differ at *P* < 0.05, *n* = 7 pens per treatment.

1 RCP0.64, 17% CP with an essential-to-total amino acid (E:T) ratio of 0.64; RCP0.68, 17% CP with an E:T ratio of 0.68; NCP, 19% CP.

No interaction was observed for FCR (*P* = 0.231), and the main effect analysis showed that reducing CP levels significantly increased FCR (*P* < 0.01), with birds on the NCP diet exhibiting the lowest FCR, which differed significantly (*P* < 0.001) from those on the RCP0.64 and RCP0.68, both of which had similar FCR values. Challenged birds exhibited significantly higher FCR (*P* < 0.001) than unchallenged birds, regardless of diet.

### 3.2 Effects of diet and NE challenge on nutrient transporter genes in the jejunum

The effects of diet and challenge on the expression of nutrient transporter genes in the jejunum are presented in Table 7. There was no interaction between diet and challenge on the expression of these genes (*P* > 0.05). The main effect analysis showed that NE challenge significantly increased the expression of *ASCT1*, *B*^*0*^*AT*, *LAT1*, and *PepT1* compared to the control (*P* < 0.05). However, *GLUT2* expression was significantly lower in challenged birds compared to their unchallenged counterparts (*P* < 0.001). Conversely, dietary treatments had no significant effect on the expression of any of the nutrient transporter genes (*P* > 0.05).

**Table 7:** Impact of diet and NE challenge on gene expression in the jejunum.

Items	Nutrient transporters						Tight junction and mucin				Immunity					Apoptosis	
	*ASCT1*	*B*^*0*^*AT*	*GLUT2*	*LAT1*	*PepT1*	*b*^*0,+*^*AT*	*TJP1*	*JAM2*	*OCLD*	*MUC2*	*IFNY*	*IL2*	*IL6*	*IgA*	*IgM*	*CAS3*	*CAS8*
**Diet**^1^																	
RCP0.64	1.43	1.07	0.97	1.01	1.24	1.06	0.96	1.12	1.04	1.00	2.38	2.02	2.99	0.90	1.37	1.09	1.21
RCP0.68	1.95	1.43	1.16	1.21	1.34	1.02	1.03	0.96	0.98	1.21	3.41	1.84	1.81	1.39	1.17	1.04	1.41
NCP	0.89	1.25	1.15	1.04	1.25	1.27	1.07	1.02	1.06	1.07	1.20	1.29	0.72	0.94	0.91	0.89	0.91
SEM	0.458	0.179	0.115	0.109	0.156	0.126	0.047	0.075	1.028	0.189	0.923	0.554	0.830	0.232	0.213	0.062	0.269
**Challenge**																	
N	0.76	0.75	1.42	0.80	0.91	1.10	1.12	1.02	1.10	1.21	0.69	0.60	0.76	1.03	1.00	0.81	0.82
Y	2.09	1.75	0.76	1.37	1.64	1.13	0.92	1.05	0.95	0.97	3.97	2.83	2.93	1.13	1.30	1.20	1.53
SEM	0.374	0.146	0.094	0.089	0.127	0.103	0.038	0.062	0.070	0.154	0.754	0.453	0.678	0.190	0.174	0.050	0.220
***P-*****value**																	
Diet	0.278	0.485	0.358	0.580	1.000	0.324	0.172	0.301	0.818	0.601	0.254	0.641	0.153	0.282	0.305	0.112	0.413
Challenge	0.020	<0.001	<0.001	<0.001	<0.001	0.899	<0.001	0.756	0.126	0.340	<0.010	<0.010	0.026	0.703	0.258	<0.001	0.033
Diet × challenge	0.138	0.160	0.374	0.912	0.370	0.145	0.776	0.906	0.527	0.179	0.200	0.919	0.582	0.902	0.221	0.467	0.185

CP = crude protein; NE = necrotic enteritis; N = no NE challenge; Y = NE challenge; SEM = standard error of mean; RCP = reduced crude protein; NCP = normal crude protein.

1 RCP0.64, 17% CP with an essential-to-total amino acid (E:T) ratio of 0.64; RCP0.68, 17% CP with an E:T ratio of 0.68; NCP, 19% CP.

### 3.3 Effects of diet and NE challenge on tight junction and mucin genes in the jejunum

The effects of diet and NE challenge on the expression of tight junction (*TJP1*, *JAM2*, and *OCLD*) and *MUC2* genes in the jejunum are shown in Table 7. No significant diet and challenge interaction was observed for any of the genes (*P* > 0.05). The main effect results showed that NE challenge significantly decreased the expression of *TJP1* compared to the control (*P* < 0.001). However, the challenge did not affect the expression of *JAM2*, *OCLD*, and *MUC2* (*P* > 0.05). Dietary treatments had no significant effect on the expression of any tight junction or mucin genes (*P* > 0.05).

### 3.4 Effects of diet and NE challenge on immunity and apoptosis genes in the jejunum

**Fig. 1:**
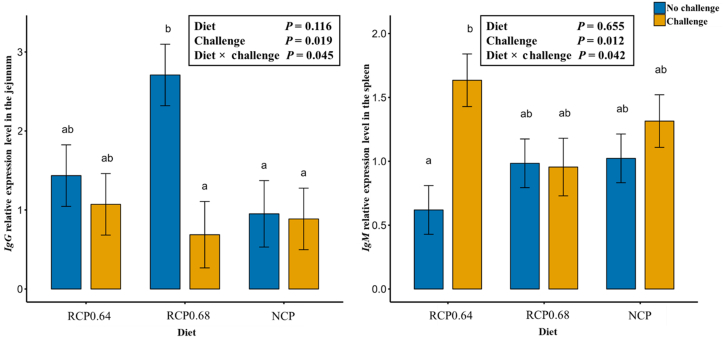
Effects of diet and NE challenge on *IgG* expression in the jejunum and *IgM* expression in the spleen. RCP0.64, 17% CP with an essential-to-total amino acid (E:T) ratio of 0.64; RCP0.68, 17% CP with an E:T ratio of 0.68; NCP, 19% CP. CP = crude protein; NE = necrotic enteritis; N = no NE challenge; Y = NE challenge; Ig = immunoglobulin; RCP = reduced crude protein; NCP = normal crude protein. Different lowercase letters above columns represent significant differences among treatments at *P* < 0.05 (*n* = 7 birds per treatment).

The effects of diet and NE challenge on the expression of immune-related genes in the jejunum are presented in Table 7 and Fig. 1. A significant interaction (*P* = 0.045) of diet and challenge was observed for *IgG* expression only (Fig. 1). Among unchallenged birds, *IgG* expression was significantly higher in the RCP0.68-fed birds than in those fed NCP (*P* = 0.045), while no significant differences were observed between RCP0.64 and either RCP0.68 or NCP-fed birds (*P* > 0.05). In challenged birds, however, *IgG* expression did not differ significantly among the dietary treatments (*P* > 0.05). The challenge exhibited higher expression levels of *IFNY*, *IL2*, and *IL6* than the control (*P* < 0.05). In contrast, dietary treatments had no significant effect on the expression of any immune-related gene (*P* > 0.05).

The effects of diet and NE challenge on the expression of cell apoptosis genes in the jejunum are presented in Table 7. No significant interaction of diet and challenge was observed for *CAS3* and *CAS8* (*P* > 0.05). The main effect results showed that the challenge significantly increased expression of *CAS3* (*P* < 0.001) and CAS8 (*P* = 0.033) compared to the control. However, dietary treatments had no significant effect on the expression of *CAS3* and *CAS8* (*P* > 0.05).

### 3.5 Effects of diet and NE challenge on immunity genes in the spleen

The effects of diet and NE challenge on the expression of immune-related genes (*IFNY*, *IL2*, *IL6*, *IgA*, *IgG*, and *IgM*) in the spleen are presented in Table 8 and Fig. 1. The main effect analysis indicates that diet significantly influenced *IL2* (*P* = 0.027) and *IL6* (*P* = 0.039) expression, where birds fed RCP0.64 exhibited similar expression to those on RCP0.68 but significantly lower expression than those on NCP (*P* < 0.05). Challenge effects revealed that challenged birds exhibited significantly higher expression of *IgA* compared to the control (*P* = 0.029).

**Table 8:** Impact of diet and NE challenge on the expression of immunity genes in the spleen.

Items		Immunity genes			
		IL2	IL6	IgA	IgG
Diet^1^	RCP0.64	0.89^a^	0.85^a^	0.76	0.93
	RCP0.68	2.71^ab^	1.37^ab^	1.39	1.24
	NCP	4.52^b^	2.98^b^	1.30	1.18
	SEM	0.939	0.622	0.306	0.203
Challenge	N	2.04	1.13	0.75	1.03
	Y	3.36	2.33	1.55	1.20
	SEM	0.768	0.508	0.250	0.166
*P-*value	Diet	0.027	0.039	0.297	0.447
	Challenge	0.216	0.101	0.029	0.398
	Diet × challenge	0.917	0.920	0.961	0.169

CP = crude protein; NE = necrotic enteritis; N = no NE challenge; Y = NE challenge; SEM = standard error of mean; RCP = reduced crude protein; NCP = normal crude protein.

Within a column, diet means without a common superscript letter differ at *P* < 0.05, *n* = 7 birds per treatment.

1 RCP0.64, 17% CP with an essential-to-total amino acid (E:T) ratio of 0.64; RCP0.68, 17% CP with an E:T ratio of 0.68; NCP, 19% CP.

A significant diet and challenge interaction (*P* = 0.042) was observed for only *IgM* expression (Fig. 1). The NE challenge significantly increased the expression of *IgM* in RCP0.64-fed birds (*P* = 0.012) but not in birds fed the other two diets (*P* > 0.05).

### 3.6 Taxonomic annotation and identification of operational taxonomic units

**Fig. 2:**
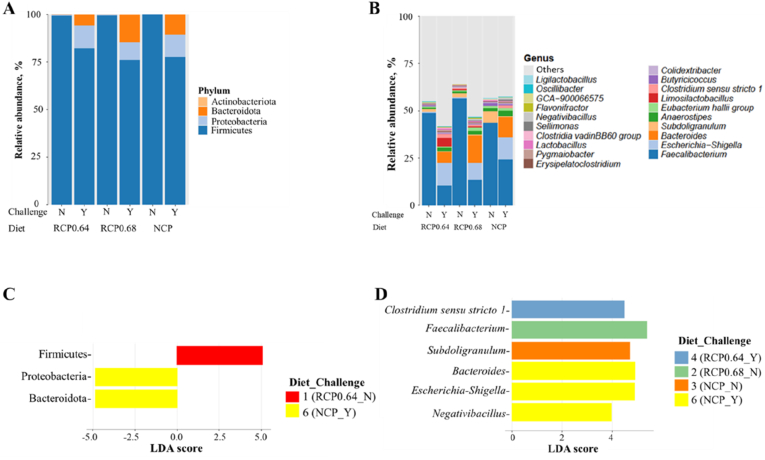
Microbial community composition and discriminant taxa in broilers fed different diets under NE challenge. (A) Relative abundance of the top phyla and (B) relative abundance of the top genera across dietary treatments and challenge status. (C–D) Linear discriminant analysis effect size (LEfSe) plots showing differentially abundant microbial taxa between groups with their linear discriminant analysis (LDA) scores at the (C) phylum level and (D) genus level. RCP0.64, 17% CP with an essential-to-total amino acid (E:T) ratio of 0.64; RCP0.68, 17% CP with an E:T ratio of 0.68; NCP, 19% CP. CP = crude protein; NE = necrotic enteritis; N = no NE challenge; Y = NE challenge; RCP = reduced crude protein; NCP = normal crude protein.

After sequence quality filtering, one sample from the RCP0.64_N group was removed due to low sequence numbers. The four most abundant phyla across all treatment groups were Firmicutes, Proteobacteria, Bacteroidota, and Actinobacteriota (Fig. 2A). Regardless of diet, the challenged birds exhibited a slightly lower abundance of Firmicutes and a higher abundance of Proteobacteria and Bacteroidota compared to the unchallenged birds. Challenged birds fed RCP0.68 had the lowest abundance of Firmicutes and the highest abundance of Bacteroidota (Fig. 2A). The dominant genera among the treatments included *Faecalibacterium*, *Escherichia*-*Shigella*, *Bacteroides*, *Subdoligranulum,* and *Anaerostipes* (Fig. 2B). *Faecalibacterium* was more abundant in unchallenged birds, while challenged birds showed elevated levels of *Escherichia-Shigella* and *Bacteroides*, regardless of dietary treatment.

### 3.7 Alpha diversity

An assessment of the diversity within each sample was performed with the Observed, Chao1, Simpson, and Shannon alpha diversity indices (Fig. S1). Descriptive statistics and Kruskal–Wallis tests for each alpha diversity metric are presented in Table S1, and no significant differences were detected among the treatment groups. Neither dietary treatment nor NE challenge significantly influenced the microbial richness and diversity of the birds.

### 3.8 Differential abundance analysis

The LEfSe analysis identified differentially abundant taxa between treatment groups at both phylum and genus levels (Fig. 2C and D). At the phylum level, Firmicutes was significantly enriched in unchallenged birds fed RCP0.64, while Proteobacteria and Bacteroidota showed significant enrichment in challenged birds fed NCP (Fig. 2C).

At the genus level, several discriminative microbial taxa associated with specific treatments were revealed (Fig. 2D). *Faecalibacterium* and *Subdoligranulum* were enriched in unchallenged birds fed diets RCP0.68 and NCP, respectively. In contrast, challenged birds fed RCP0.64 demonstrated significant enrichment of *Clostridium* *sensu stricto 1*, while challenged NCP-fed birds showed increased abundance of *Bacteroides*, *Escherichia-Shigella*, and *Negativibacillus*.

### 3.9 Beta diversity

**Fig. 3:**
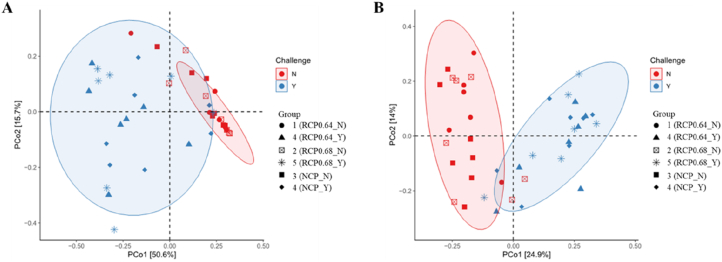
Principal coordinates analysis (PCoA) plots showing microbial beta diversity. (A) Weighted UniFrac. (B) Unweighted UniFrac distance matrices. Each point represents an individual sample, shapes denote treatment groups, and colors represent challenge status. Ellipses indicate the 95% confidence intervals for each challenge group. RCP0.64, 17% CP with an essential-to-total amino acid (E:T) ratio of 0.64; RCP0.68, 17% CP with an E:T ratio of 0.68; NCP, 19% CP. CP = crude protein; NE = necrotic enteritis; N = no NE challenge; Y = NE challenge; RCP = reduced crude protein; NCP = normal crude protein.

The PCoA, based on weighted (Fig. 3A) and unweighted (Fig. 3B) UniFrac distances, was used to evaluate beta diversity across treatment groups. Both distance metrics revealed a clear separation between challenged and unchallenged birds, suggesting distinct microbial community compositions.

The PERMANOVA analysis clarified the distinct microbial composition between challenged and unchallenged birds. For weighted UniFrac, significant differences were observed between unchallenged and challenged groups (e.g., RCP0.64_N vs. RCP0.64_Y, RCP0.68_N vs. RCP0.68_Y, and NCP_N vs. NCP_Y) (Table 9). Similarly, unweighted UniFrac comparisons revealed significant differences between challenged and unchallenged birds (e.g., RCP0.64_N vs. RCP0.64_Y, NCP_N vs. NCP_Y) (Table 10).

**Table 9:** PERMANOVA results based on Weighted UniFrac distances.

Paired comparison^1^	F	*R*^2^	*P*-value	Adjusted *P*-value	Significance
2 vs. 3	0.520	0.040	0.640	0.740	
2 vs. 1	0.590	0.050	0.690	0.740	
2 vs. 4	7.760	0.390	<0.001	0.020	∗
2 vs. 5	6.620	0.360	0.010	0.020	∗
2 vs. 6	5.010	0.300	0.010	0.020	∗
3 vs. 1	0.580	0.050	0.660	0.740	
3 vs. 4	7.140	0.370	<0.001	0.020	∗
3 vs. 5	6.110	0.340	0.010	0.020	∗
3 vs. 6	4.170	0.260	0.020	0.040	∗
1 vs. 4	5.270	0.320	<0.001	0.020	∗
1 vs. 5	4.570	0.290	0.010	0.020	∗
1 vs. 6	3.250	0.230	0.030	0.050	∗
4 vs. 5	0.470	0.040	0.810	0.810	
4 vs. 6	1.390	0.100	0.240	0.360	
5 vs. 6	0.910	0.070	0.430	0.590	

PERMANOVA = permutational analysis of variance; CP = crude protein; N = no necrotic enteritis challenge; Y = necrotic enteritis challenge; ∗ = adjusted *P*-value < 0.05.

1 Numbers (1–6) correspond to the treatment combinations defined in Table 1.

**Table 10:** PERMANOVA results based on Unweighted UniFrac distances.

Paired comparison^1^	F	*R*^2^	*P*-value	Adjusted *P*-value	Significance
2 vs. 3	0.890	0.070	0.540	0.680	
2 vs. 1	0.930	0.080	0.530	0.680	
2 vs. 4	3.180	0.210	0.010	0.010	∗
2 vs. 5	2.800	0.190	<0.001	0.010	∗∗
2 vs. 6	2.750	0.190	<0.001	0.010	∗∗
3 vs. 1	1.110	0.090	0.290	0.440	
3 vs. 4	4.390	0.270	<0.001	0.010	∗∗
3 vs. 5	4.040	0.250	<0.001	0.010	∗∗
3 vs. 6	4.130	0.260	<0.001	0.010	∗∗
1 vs. 4	3.840	0.260	<0.001	0.010	∗∗
1 vs. 5	2.820	0.200	<0.001	0.010	∗∗
1 vs. 6	3.140	0.220	<0.001	0.010	∗∗
4 vs. 5	0.800	0.060	0.640	0.740	
4 vs. 6	0.640	0.050	0.820	0.880	
5 vs. 6	0.610	0.050	0.890	0.890	

PERMANOVA = permutational analysis of variance; CP = crude protein; NE = necrotic enteritis; N = no NE challenge; Y = NE challenge; ∗ = adjusted *P*-value < 0.05; ∗∗ = adjusted *P* < 0.001.

1 Numbers (1–6) correspond to the treatment combinations defined in Table 1.

**Fig. 4:**
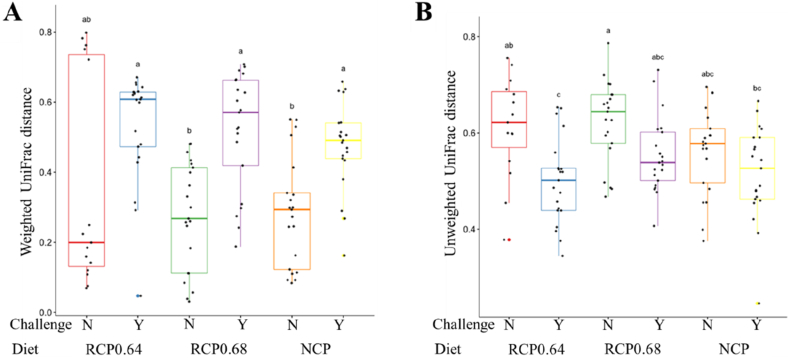
Boxplots showing beta diversity comparisons based on UniFrac distances among treatment groups. (A) Weighted UniFrac distances. (B) Unweighted UniFrac distances. RCP0.64, 17% CP with an essential-to-total amino acid (E:T) ratio of 0.64; RCP0.68, 17% CP with an E:T ratio of 0.68; NCP, 19% CP. CP = crude protein; NE = necrotic enteritis; N = no NE challenge; Y = NE challenge; RCP = reduced crude protein; NCP = normal crude protein. Different lowercase letters above columns represent significant differences among treatments at *P* < 0.05.

To further explore these group-level differences, Dunn’s Kruskal–Wallis pairwise comparisons of Weighted and Unweighted UniFrac distances were visualized using boxplots (Fig. 4). In the Weighted UniFrac analysis (Fig. 4A), challenged birds in RCP0.68_Y and NCP_Y had significantly greater distances than their unchallenged counterparts RCP0.68_N and NCP_N. Unweighted UniFrac distances (Fig. 4B) showed that unchallenged birds had significantly greater distances than challenged ones only in RCP0.64-fed birds.

## 4 Discussion

This study investigated the effects of varying E:T ratios in RCP diet on growth performance, gene expression patterns, and cecal microbiota in broiler chickens, particularly under NE challenge. This study tested the hypothesis that optimal E:T RCP diets could positively impact birds at a level similar to that of standard CP-fed birds and mitigate the detrimental effects of NE on broiler performance, intestinal function, and gut microbiota. This hypothesis is supported by the fact that in unchallenged birds, RCP0.64 yielded WG and FI comparable to the control and superior to the RCP0.68-fed birds. Under NE challenge, the RCP0.64 diet maintained higher FI compared to the control diet. Moreover, the RCP0.64 diet partially preserved gut health during NE challenge by modulating immune responses (*IgG* and *IgM*) and microbial communities. Therefore, the hypothesis that an optimized 0.64 E:T RCP diet can sustain WG and FI in healthy birds and offer partial protection during NE challenge was accepted.

The study identified a significant diet × NE challenge interaction for WG and FI, but not for FCR. Among unchallenged birds, those fed RCP0.64 and NCP diets exhibited significantly higher WG compared to birds fed RCP0.68, with no significant difference observed between RCP0.64 and NCP-fed birds. This suggests that an optimized E:T ratio of 0.64 can effectively compensate for RCP levels, maintaining performance (WG and FI) outcomes comparable to a standard protein diet. These findings are consistent with previous findings that recommended an optimal E:T ratio of 0.60 (excluding Tyr in EAA) or 0.64 (including Tyr in EAA) to support growth under reduced protein diets (Asiamah et al., 2025; Musigwa et al., 2024). Asiamah et al. (2025) further reported that birds fed the 0.64 E:T reduced protein diet exhibited upregulation of genes involved in the PPAR signaling pathway, which enabled them to utilize fats as alternative energy sources to maintain body weight despite protein reduction. This suggests that birds on RCP0.64 in the present study, which showed similar WG to NCP-fed birds, may have activated the PPAR signaling pathway in this study. The dietary advantage of RCP0.64 on WG was nullified by the NE challenge, indicating that the NE stress may overshadow the benefits of AA optimization in RCP diets. This observation aligns with earlier studies demonstrating that NE challenge diminishes the growth-promoting potential of dietary interventions during the d 10 to 24 grower stage of broiler chickens (Kumar et al., 2021b). The FI patterns closely mirrored WG trends. In unchallenged birds, RCP0.64-fed birds consumed significantly more feed than those on RCP0.68, while FI was comparable between RCP0.64 and NCP. The improved FI and WG in RCP0.64-fed healthy birds may reflect enhanced nutrient utilization, as shown in earlier studies (Asiamah et al., 2025). Under challenge conditions, RCP0.64-fed birds maintained the highest FI, suggesting these birds may have better appetite or coping capacity during stress. Nevertheless, this did not translate into superior WG, possibly due to reduced nutrient digestibility or absorption under NE stress (Kumar et al., 2021a). The main effect analysis showed that the RCP0.64 diet increased FCR compared to the NCP control diet, consistent with earlier findings (Asiamah et al., 2025). This finding agrees with reports that broilers can achieve efficient nutrient utilization by increasing FI, which enhances growth rates but may compromise FCR, especially on RCP diets (Barekatain et al., 2023; Belloir et al., 2017; Musigwa et al., 2024). Likewise, NE challenge as a main effect significantly increased FCR compared to the unchallenged birds, supporting previous findings reported elsewhere (Gharib-Naseri et al., 2021; Keerqin et al., 2021). The poor performance observed in birds fed RCP0.68 in both challenged and unchallenged groups may be due to an imbalance of EAA and TAA, as elucidated in earlier studies (Waldroup et al., 1976). Overall, these results highlight that while a 0.64 E:T optimized RCP diet maintains WG and FI similar to standard protein diets under healthy conditions, its benefits may be diminished under NE stress. NE-challenged birds, regardless of diet, had significantly lower WG and FI, and higher FCR than unchallenged birds, consistent with previous findings in Cobb 500, Athens Canadian Random Bred, and Ross 308 broilers (Gharib-Naseri et al., 2021; Goo et al., 2024, 2023; Keerqin et al., 2021).

Nutrient transporters across the plasma membrane in the small intestine play a crucial role in regulating AA and energy absorption, which are essential for growth, feed efficiency, immune function, and disease resistance in animals (Bröer, 2008; Gharib-Naseri et al., 2021). The main effects of this study found that NE challenge significantly influenced the expression of several nutrient transporter genes in the jejunum. Specifically, birds under NE challenge showed increased expression of *ASCT1*, *B*^*0*^*AT*, *LAT1*, and *PepT1*, while *GLUT2* expression was significantly reduced. The higher expression of *ASCT1* in challenged birds observed in this study is consistent with findings in coccidiosis-challenged birds, where *ASCT1* was upregulated as a compensatory mechanism, which supports AA (Thr, Cys, Ser, and Ala) transport and uptake for epithelial recovery (Casterlow et al., 2011). *ASCT1* also transports Glu, a key energy source for epithelial cells, highlighting its role in stress recovery and cellular repair (Casterlow et al., 2011). Similarly, the increased expression of *B*^*0*^*AT* in the challenged birds likely enhanced the absorption of critical AA such as Met, Leu, Ile, Val, Thr, Cys, Phe, and Arg to support growth and immune defense (Castro and Kim, 2020; Wang and Zou, 2020). The higher expression of *LAT1* in this study aligns with findings from *Eimeria*-challenged chickens, where higher *LAT1* was associated with increased AA demand during intestinal inflammation (Fetterer et al., 2014; Teng et al., 2021). These authors suggested that the infection may deplete essential AA in infected cells by upregulating AA transporters at the basolateral membrane and downregulating nutrient transporters at the brush border membrane. This confirms the higher expression of the AA transporters (*ASCT1*, *B*^*0*^*AT*, and *LAT1*) and lower expression of the nutrient transporter (*GLUT2*) in the challenged birds in this study. The lower expression of *GLUT2*, a nutrient transporter responsible for the absorption of glucose, galactose, mannose, and fructose (Kellett et al., 2008; Mueckler and Thorens, 2013), may impair carbohydrate transport and absorption. This could reduce energy availability, partly contributing to compromised growth and immunity in challenged birds (Gharib-Naseri et al., 2021; Goo et al., 2023). *PepT1*, a peptide transporter responsible for the uptake of dipeptides and tripeptides into enterocytes, exhibited increased expression in challenged birds in this study, further supporting enhanced nutrient absorption under stress (Smith et al., 2013). No significant differences in transporter gene expression were observed across the dietary treatments, indicating that the diets did not influence the intestinal transporter transcription of these genes. This observation aligns with previous studies in which neither the standard nor reduced protein diet had a significant impact on transporter gene expression when AA requirements were met (England et al., 2024). These results suggest that NE challenge exerts a dominant effect on nutrient transporter gene expression in the jejunum, potentially as a host strategy to counteract reduced nutrient absorption due to intestinal damage. The lack of dietary effects further implies that transporter regulation under pathogenic stress may be more immune-driven than nutrient-driven.

The expression levels of tight junction proteins and mucin genes are indicators of epithelial barrier function. In this study, NE challenge significantly suppressed the expression of *TJP1*, whereas *JAM2*, *OCLD*, and *MUC2* remained unaffected. Neither dietary interventions using optimized E:T ratios nor diet × challenge interaction had a significant effect. The downregulation of *TJP1* in challenged birds is consistent with previous findings and reflects compromised intestinal barrier function (Gharib-Naseri et al., 2021; Keerqin et al., 2021). Since *TJP1* plays a central role in regulating paracellular permeability and maintaining intestinal integrity, its reduced expression likely indicates epithelial disruption resulting from NE pathology. This finding aligns with the observed impairment in performance and nutrient transporter gene regulation in challenged birds. However, the absence of any compensatory dietary effect on *TJP1* expression suggests that the diets used in this study may not modulate the transcriptional response of tight junctions under NE stress. In contrast, *MUC2*, which encodes the primary mucin glycoprotein responsible for epithelial protection (McGuckin et al., 2011), was not significantly altered by NE challenge. The dissociation between *TJP1* and *MUC2* responses indicates that NE pathogenesis in this study may have primarily targeted the physical barrier rather than the chemical defense system. Overall, the reduced *TJP1* expression without a corresponding upregulation of protective genes such as *MUC2* highlights that the NE challenge has a greater effect on epithelial integrity than dietary protein level or AA balance, at least in the jejunum.

The immune response of broiler chickens to dietary modulation and pathogen challenge is critical for maintaining health and performance. In this study, NE challenge induced a strong immune reaction marked by significant upregulation of key cytokines and immunoglobulins, whereas dietary interventions exerted selective immunomodulatory effects. These results provide insights into how nutrition and infection interact to shape host immunity during NE. This study revealed that NE challenge significantly increased the expression of immune-related genes (*IL2*, *IFNY*, and *IL6*) in the jejunum, which is consistent with previous reports linking NE infection to increased cytokine activity (Criado-Mesas et al., 2021; Goo et al., 2023). *IL2* plays an important role in T-cell proliferation and natural killer cell activation (Susta et al., 2015), and has been shown to induce *IFNY* secretion, a key mediator of the innate and adaptive immune system (Puddu et al., 2005; Tau and Rothman, 1999). *IFNY*, in turn, stimulates the production of *IL6,* contributing to the pathogenesis of inflammatory diseases (Ishimaru et al., 2013). This chain of cytokine activation may explain the simultaneous upregulation of *IL2*, *IFNY*, and *IL6* in the jejunum of the challenged birds. Notably, the upregulation of *IFNY* is related to the downregulation of tight junction proteins such as *TJP1* (Scharl et al., 2009), which aligns with reduced *TJP1* expression in challenged birds in this study. Gharib-Naseri et al. (2020) attributed the increase in tight junction proteins to the downregulation of *IFNY* as the pathogen load in the gut decreased with probiotic supplementation. Significant dietary effects were observed in the spleen, where birds fed RCP0.64 presented significantly lower *IL2* and *IL6* expression than those fed NCP. These findings suggest that an optimal 0.64 E:T balance may decrease systemic inflammation, as dietary protein and AA can modulate immune gene expression (Kamely et al., 2020; Li et al., 2007). While the cytokine response was primarily challenge-driven, notable diet × challenge interactions were evident for immunoglobulins. Challenged birds fed the RCP0.64 diet showed increased expression of *IgG* in the jejunum and *IgM* in the spleen, indicating that the RCP0.64 diet may support humoral immune function under stress. *IgG* is a major class of serum antibodies, whereas *IgM* is a frontline defense during infection (Carlander et al., 2010; Liu et al., 2019). Previous reports have shown that protein and AA supplementation can increase antibody production during disease (Alagawany et al., 2020). The NE challenge significantly increased apoptotic genes *CASP3* and *CASP8* expression, reflecting epithelial cell death and tissue damage, as previously reported (Gharib-Naseri et al., 2020). These caspases are critical in mediating programmed cell death and inflammatory signaling (McIlwain et al., 2013). Overall, NE challenge activated strong cytokine and apoptotic responses in broiler chickens, yet dietary intervention with a 0.64 E:T optimized RCP diet offered potential immunomodulatory benefits. This dietary strategy may help maintain immune competence without exacerbating inflammation during NE outbreaks.

The gut microbiota plays a vital role in nutrient digestion, immune modulation, and the performance of broiler chickens. In this study, diet and NE challenge influenced microbial composition and structure, with notable implications for performance, immunity, and gut health. The PCoA based on weighted and unweighted UniFrac distances revealed clear clustering by challenge status. Unchallenged birds exhibited tighter clustering, suggesting a more stable and uniform microbial community, whereas challenged birds displayed greater dispersion, indicating microbial variability, disruption, and dysbiosis. At the phylum level, Firmicutes, Bacteroidota, Proteobacteria, and Actinobacteriota dominated across all treatments, similar to previous reports in broiler NE models (Bortoluzzi et al., 2019; Miska et al., 2023). Firmicutes were more abundant among unchallenged birds, with a differential abundance in RCP0.64-fed birds, which coincided with improved WG and FI. Firmicutes are essential for energy harvesting, short-chain fatty acid production, and nutrient absorption, and their relatively high abundance has been positively linked to feed efficiency and WG in chickens (Adewole and Akinyemi, 2021; Chang et al., 2023; He et al., 2023). In contrast, NE challenge decreased Firmicutes and increased Bacteroidota and Proteobacteria, particularly in birds fed RCP0.68 and NCP. These shifts are characteristic of gut dysbiosis and have been associated with intestinal inflammation and metabolic dysfunction (Shin et al., 2015; Stojanov et al., 2020). Although it is generally associated with carbohydrate metabolism and short-chain fatty acid production (Hooper et al., 2002; Thomas et al., 2011), Bacteroidota can dominate under stress and impair microbial balance, particularly when elevated alongside Proteobacteria. The increased abundance of Proteobacteria, often linked to microbiota imbalance and metabolic dysfunction (Shin et al., 2015), indicates impaired microbial equilibrium in challenged birds. These microbial changes likely contributed to the poor performance and higher expression of immune-related (*IFNY*, *IL2*, and *IL6*) and apoptotic genes (*CAS3* and *CAS8*) in the NE-challenged birds in this study, reflecting immune activation and tissue damage in response to microbial imbalance. At the genus level, the unchallenged birds harbored a greater abundance of beneficial taxa, such as *Faecalibacterium* and *Subdoligranulum*. These genera are integral to butyrate and acetic acid production, contributing to intestinal barrier function, energy metabolism, and anti-inflammatory regulation (Obianwuna et al., 2024; Rossi et al., 2016; Van Hul et al., 2020). Lee et al. (2017) revealed that *Faecalibacterium* is associated with increased body weight as observed in the unchallenged birds. Thus, reduced abundance of these genera in challenged birds may reflect impaired mucosal function and energy absorption. Interestingly, *Faecalibacterium* was enriched in unchallenged birds fed RCP0.68, while *Subdoligranulum* was prominent in those fed NCP, likely influenced by higher levels of dietary AA and CP, respectively (Jianfei et al., 2019). Another notable genus highly abundant in the RCP0.64-challenged birds was *Limosilactobacillus*, which is known to modulate gut microbiota, enhance the production of anti-inflammatory cytokines, and promote the secretion of antimicrobial molecules (Abuqwider et al., 2022). Previous studies have shown that *Limosilactobacillus* supplementation alleviates intestinal villus damage, increases serum *IgM* levels, and suppresses proinflammatory cytokine secretion in broilers under NE and *Salmonella* challenge (Guo et al., 2023; Šefcová et al., 2023). Similar to these findings, challenged birds fed RCP0.64 with the highest abundance of *Limosilactobacillus* had relatively increased expression of *IgG* in the jejunum and *IgM* in the spleen, suggesting enhanced immune response against NE severity and higher FI compared to the other challenged birds. Conversely, *Escherichia-Shigella*, *Bacteroides*, and *C*. *sensu stricto 1* were enriched in NE-challenged birds, particularly in those fed RCP0.64 and NCP. These genera are commonly associated with gut inflammation and mucosal disruption (Hu et al., 2021; Yang et al., 2021). *Escherichia-Shigella* co-occurs with NE pathology and correlates positively with NE severity and cytokine upregulation in broiler chickens (Li et al., 2017; Yang et al., 2021). This explains the elevated abundance of *Escherichia-Shigella* observed in all challenged groups. This may also explain why the cytokines in this study were highly expressed in the NE-challenged birds. The *Bacteroides* genus, a member of the *Bacteroidaceae*, capable of metabolizing carbohydrates and polysaccharides to promote nutrient absorption (Yang et al., 2022), can dominate under stress and compromise mucosal integrity. The greater abundance of *Bacteroides* in NE challenged birds could be related to their poorer performance, as shown by England et al. (2024), who reported greater *Bacteroides* in low-performing female birds. *C*. *sensu stricto 1* is an opportunistic pathogenic bacterium that can cause intestinal inflammation and decrease the content of short-chain fatty acids (Hu et al., 2021). A study in broiler chickens revealed that the overgrowth of *C. sensu stricto 1* was associated with NE, contributing to the development of the disease (Yang et al., 2019). They further explained that *C. sensu stricto 1* contains *C*. *perfringens* and other *Clostridium* species, generally perceived as pathogenic. In this study, the enriched abundance of *C. sensu stricto 1* in the RCP0.64-challenged birds may have contributed to the development of NE in these birds, potentially offsetting some of the physiological benefits associated with the optimized 0.64 E:T RCP diet. The differential abundance of these inflammatory taxa in RCP0.64 and NCP-fed challenged birds may be due to the higher amount of CP and AA in the diet. This may cause excessive undigested protein substrates, which can promote the growth of pathogenic bacteria and exacerbate gut inflammation (Jianfei et al., 2019). Together, these findings indicate that the gut microbiota composition was significantly influenced by NE challenge and, to a lesser extent, by diet. Birds fed the RCP0.64 diet under NE challenge maintained more favorable microbial profiles, characterized by higher Firmicutes and *Limosilactobacillus* and lower inflammatory taxa. This suggests that the RCP0.64 diet may buffer the negative effects of NE-induced dysbiosis, supporting microbial stability and maintaining critical gut functions.

## 5 Conclusion

This study reveals that the 0.64 E:T optimized RCP diet maintained WG and FI comparable to those on the control diet under healthy conditions. Across all diets, jejunal expression of genes related to nutrient transport, barrier function, and immunity was similar, while proinflammatory cytokines (*IL2* and *IL6*) remained lowest in the spleen of RCP0.64 birds. The NE challenge significantly impaired performance, disrupted microbial composition, and altered gene expression associated with nutrient transport, barrier function, and immunity. Under challenged conditions, birds fed the RCP0.64 diet yielded higher FI compared to the control diet and exhibited a more moderate immune response and microbial profile. The RCP0.64-challenged birds exhibited higher abundance of beneficial microbial taxa, such as Firmicutes and *Limosilactobacillus* and increased expression of immunoglobulins (*IgG* in the jejunum and *IgM* in the spleen) compared to RCP0.68 and NCP-challenged birds, suggesting enhanced physiological and immune resilience. Although the RCP0.64 diet could not completely mitigate the adverse effects of NE, it helped reduce their severity. These findings suggest that an optimized 0.64 E:T RCP diet provides a promising strategy to support broiler health and performance comparable to normal CP diets, even under NE challenge, thereby contributing to sustainable poultry production with reduced reliance on high-protein diets. Future studies could explore combining this dietary strategy with feed additives, such as probiotics, prebiotics, organic acids, and phytobiotics, to further mitigate the adverse effects of NE.

## Credit Author Statement

**Collins A. Asiamah:** Writing – review & editing, Writing – original draft, Visualization, Software, Methodology, Investigation, Formal analysis, Data curation, Conceptualization. **Sosthene Musigwa:** Writing – review & editing, Visualization, Validation, Methodology, Investigation, Data curation, Conceptualization. **Sarbast K. Kheravii:** Writing – review & editing, Validation, Methodology. **Alip Kumar:** Writing – review & editing, Validation, Methodology. **Yadav S. Bajagai:** Writing – review & editing, Visualization, Validation, Software, Methodology, Formal analysis. **Sara de las Heras-Saldana:** Writing – review & editing, Visualization, Validation, Supervision. **Shu-Biao Wu:** Writing – review & editing, Visualization, Validation, Supervision, Resources, Project administration, Methodology, Investigation, Funding acquisition, Conceptualization.

## Declaration of competing interest

We declare that we have no financial and personal relationships with other people or organizations that can inappropriately influence our work, and there is no professional or other personal interest of any nature or kind in any product, service and/or company that could be construed as influencing the content of this paper.
