# Supplementary material for: Optimized amino acid ratios in reduced crude protein diets sustain broiler performance, gut health, and resilience to necrotic enteritis
Source: Anim Nutr. 2026 Mar 6;25:476–91. doi: 10.1016/j.aninu.2025.11.011 (PMC13089080; doi:10.1016/j.aninu.2025.11.011)
Supplement: Multimedia component 1 [file mmc1.docx]

**Table S1** Descriptive statistics of alpha diversity indices across treatment groups.

| Treatments^1^ | *n* | Observed | Chao1 | Shannon | Simpson |
| --- | --- | --- | --- | --- | --- |
| RCP0.64_N | 6 | 58.50 | 58.63 | 3.69 | 0.97 |
| RCP0.64_Y | 7 | 58.71 | 58.71 | 3.68 | 0.97 |
| RCP0.68_N | 7 | 47.86 | 47.86 | 3.55 | 0.96 |
| RCP0.68_Y | 7 | 53.71 | 53.71 | 3.60 | 0.96 |
| NCP_N | 7 | 61.00 | 61.00 | 3.81 | 0.97 |
| NCP_Y | 7 | 58.43 | 58.43 | 3.73 | 0.97 |
| SEM |  | 5.922 | 5.934 | 0.127 | 0.006 |
| *P*-value^2^ |  | 0.611 | 0.607 | 0.580 | 0.426 |

CP = crude protein; N = no necrotic enteritis challenge; Y = necrotic enteritis challenge; SEM = standard error of mean; RCP = reduced crude protein; NCP = normal crude protein.

^1^RCP0.64, 17% CP with an E:T ratio of 0.64; RCP0.68, 17% CP with an E:T ratio of 0.68; NCP, 19% CP.

^2^*P*-values are from the Kruskal-Wallis test across the six treatment groups.

**Table S2** Abbreviations and full names of genes.

| Abbreviations | Full names |
| --- | --- |
| *ASCT1* | Alanine, serine, cysteine, and threonine transporter-1 |
| *b*0,+AT | b0,+Amino acid transporter |
| *B*0AT | Neutral amino acid transporter |
| *CASP3* | Caspase 3 |
| *CASP8* | Caspase 8 |
| *GLUT2* | Glucose transporter-2 |
| *IFNY* | Interferon-gamma |
| *IgA* | Immunoglobulin A |
| *IgG* | Immunoglobulin G |
| *IgM* | Immunoglobulin M |
| *IL2* | Interleukin 2 |
| *IL6* | Interleukin 6 |
| *JAM2* | Junctional adhesion 2 |
| *LAT1* | L-Amino acid transporter-1 |
| *MUC2* | Mucin 2 |
| *OCLD* | Occludin |
| *PepT1* | Peptide transporter 1 |
| *PPAR* | Peroxisome proliferator-activated receptor |
| *RPL4* | Ribosomal protein L4 |
| *SDHA* | Succinate dehydrogenase complex flavoprotein subunit A |
| *TJP1* | Tight junction protein 1 |
| *β-ACT* | Beta-actin |


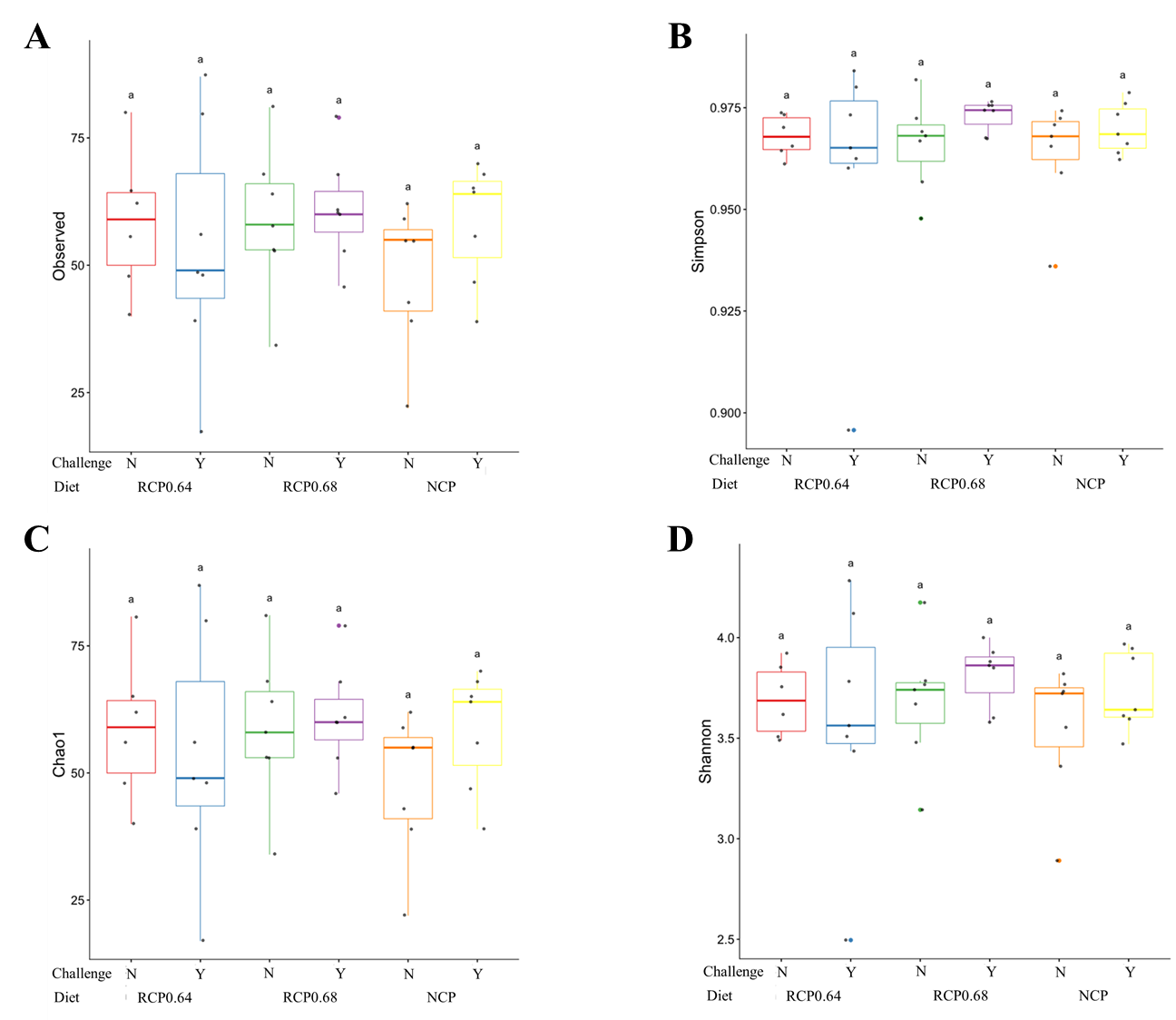


**Fig. S1** Boxplots of alpha diversity indices across treatment groups. (A) Observed. (B) Shannon. (C) Chao1. (D) Simpson. Each box represents the distribution of diversity values for each group, and dots represent individual sample values. RCP0.64, 17% CP with an E:T ratio of 0.64; RCP0.68, 17% CP with an E:T ratio of 0.68; NCP, 19% CP. CP = crude protein; NE = necrotic enteritis; N = no NE challenge; Y = NE challenge; RCP = reduced crude protein; NCP = normal crude protein.
